# Facilitators, Barriers, and Potential Impacts of Implementation of e-Pharmacy in India and its Potential Impact on Cost, Quality, and Access to Medicines: Scoping Review

**DOI:** 10.2196/51080

**Published:** 2024-10-09

**Authors:** Aditi Apte, Heber Rew Bright, Sandeep Kadam, Thambu David Sundarsanam, Sujith J Chandy

**Affiliations:** 1 KEM Hospital Research Centre Pune India; 2 Department of Pharmacy Christian Medical College Vellore India; 3 Department of Pediatrics and Neonatology KEM Hospital Pune India; 4 Department of Medicine Christian Medical College Vellore India; 5 Department of Pharmacology and Clinical Pharmacology Christian Medical College Vellore India

**Keywords:** online pharmacy, internet pharmacy, telepharmacy, ePharmacy, prescribing systems, drug prescribing, prescriptions, medications

## Abstract

**Background:**

e-Pharmacy can potentially solve problems related to the quality of services and products, cost, and access to medicines in low- and middle-income countries. This review aims to understand the facilitators and barriers to the implementation of e-pharmacy in India.

**Objective:**

This scoping review aimed (1) to understand the facilitators and barriers to the use of e-pharmacy in India and (2) to estimate the potential for e-pharmacy in India for improving access to medication, improving the quality of services and medicines, and decreasing costs of medications.

**Methods:**

All published and gray literature from July 1, 2011, to June 30, 2021, relating to e-pharmacy, was searched from MEDLINE, Scopus, ProQuest, and Google using a systematic search strategy.

**Results:**

In total, 1464 titles and abstracts were screened, of which 47 full-texts were included in the review. e-Pharmacy can potentially improve access to medications for remote areas, and old and debilitated individuals. e-Pharmacies can enable lean supply chain management, lower cost, and allow easy tracking of dispensed medicines. There is potential for integration of e-pharmacy services into the national program of Bhartiya Jan Aushadhi Pariyojana. However, the country is not adequately regulated to prevent the growth of illicit e-pharmacies. Lack of global accreditation and internet coverage, digital literacy, and transnational access are other challenges.

**Conclusions:**

E-pharmacy has the potential to improve universal health coverage in India by improving access to medicines and lowering the overall cost of health care. However, future growth will need specific regulations and accreditation mechanisms.

**Trial Registration:**

Open Science Forum; https://doi.org/10.17605/OSF.IO/6R9YQ

## Introduction

e-Pharmacy has been defined as the “business of distribution or sale, stock, exhibit, or offer for sale of medicines through the web portal or any other electronic mode” [1]. In recent times, there has been a substantial increase in the use of e-pharmacy in India due to the growth of the e-commerce sector, increasing internet penetration, and the use of smartphones [2]. During the COVID-19 pandemic, health care in India witnessed a substantial transformation due to the wide use of telemedicine services, including online prescriptions and e-pharmacies [3]. The Indian e-pharmacy market stood at US $344.78 million in 2021 and is expected to witness 21.28% growth by 2027 [4]. Soon, the e-pharmacy model may account for 5%-15% of the total medicine sales in India and thus may contribute to health care substantially along with the traditional brick-and-mortar pharmacies [5].

e-Pharmacy platforms offer a convenient, doorstep delivery of medicines to consumers, resulting in a rising global demand for the model [6]. e-Pharmacy can, therefore, prove to be an important tool in the armamentarium of Indian health policy makers to improve universal health coverage [7]. On the other hand, the growth of rogue e-pharmacies is a significant concern worldwide, especially in low- and middle-income countries (LMICs), which often lack specific regulations for the online sale of medicines [8,9]. The proliferation of e-pharmacy in India so far has been largely beyond existing regulatory mechanisms with issues related to the quality of medicines and dispensing errors [10,11,12].

There is a lack of systematic evidence on the status of e-pharmacy in India and its potential in the near future to improve health access. The present scoping review was undertaken to (1) understand the facilitators and barriers to the use of e-pharmacy in India and (2) estimate the potential for e-pharmacy in India for improving access to medication, improving the quality of services and medicines, and decreasing costs of medications.

## Methods

### Protocol Registration

The review was carried out in accordance with the PRISMA-ScR (Preferred Reporting Items for Systematic Reviews and Meta-Analyses extension for Scoping Reviews) guidelines [13]. The protocol was registered with the Open Science Framework (OSF).

### Search Strategy and Selection Criteria

MEDLINE, Scopus, ProQuest, and Google were searched for scientific literature, and Google and ProQuest were searched for gray literature published from July 1, 2011, to June 30, 2021, which were written in English or Hindi were included. Relevant literature published from June 30, 2021, till September 30, 2022, has been added after the completion of the initial search and data extraction to keep the readers updated about recent developments. Original papers, review papers, newspaper articles, reports from government or nongovernmental organizations, guidelines and policy documents, newsletters, web pages, blogs, and PowerPoint (Microsoft Corp) presentations were included.

Inclusion criteria were original papers and other publications, as mentioned above, related to the technologies used for e-pharmacy; regulations for controlling e-pharmacy in India and other countries; the impact of e-pharmacy on access, supply chain management, quality of services, availability of essential medicines, and cost (especially in the context of primary health care). Global literature on facilitators and barriers to e-pharmacy deemed important to the Indian context was included. Literature from developed countries that did not fit in the context of developing nations was excluded from the review. Literature related to telemedicine without any relevance to e-pharmacy was excluded from the review.

A 3-step search strategy as defined in the standard Joanna Briggs Institute (JBI) systematic reviews was used for this purpose. As a first step, a limited search of the relevant databases was done, following which analysis of the key terms used in the title, abstract, or index was done. In step 2, keywords were extracted from the literature identified during the first step. Variants and combinations of search terms relating to these keywords (e-pharmacy, facilitators and barriers, access, quality, cost, and regulations) were used for searching (Table S1 in Multimedia Appendix 1). In step 3, reference lists of the identified articles were searched for relevant references.

### Publication Selection

The title and abstract of the available search results were screened using DistillerSR (Distiller Inc) simultaneously but independently by 2 authors (HRB and SK) based on the given eligibility criteria. In case of any uncertainty regarding the inclusion of the article, a third author (AA) was consulted. The full texts were then procured for the selected number of articles, which were screened independently by 2 authors (AA and HRB), and for any conflicts between the 2 authors, a third author (SJC) was consulted.

### Data Charting

Data charting for the selected full-text articles was carried out by 3 authors (AA, SK, and HRB) using data collection forms generated using DistillerSR software. Charting performed by one author was verified by one more author. The data extracted included information on the study characteristics (author, year of publication, country, type of publication, and focus group) and outcomes (Technology used for e-pharmacy, advantages, disadvantages, regulations, access or availability, cost, affordability, and quality of service). The final datasheet was exported as an Excel file from the software, which was used for the compilation of results.

## Results

**Figure 1:**
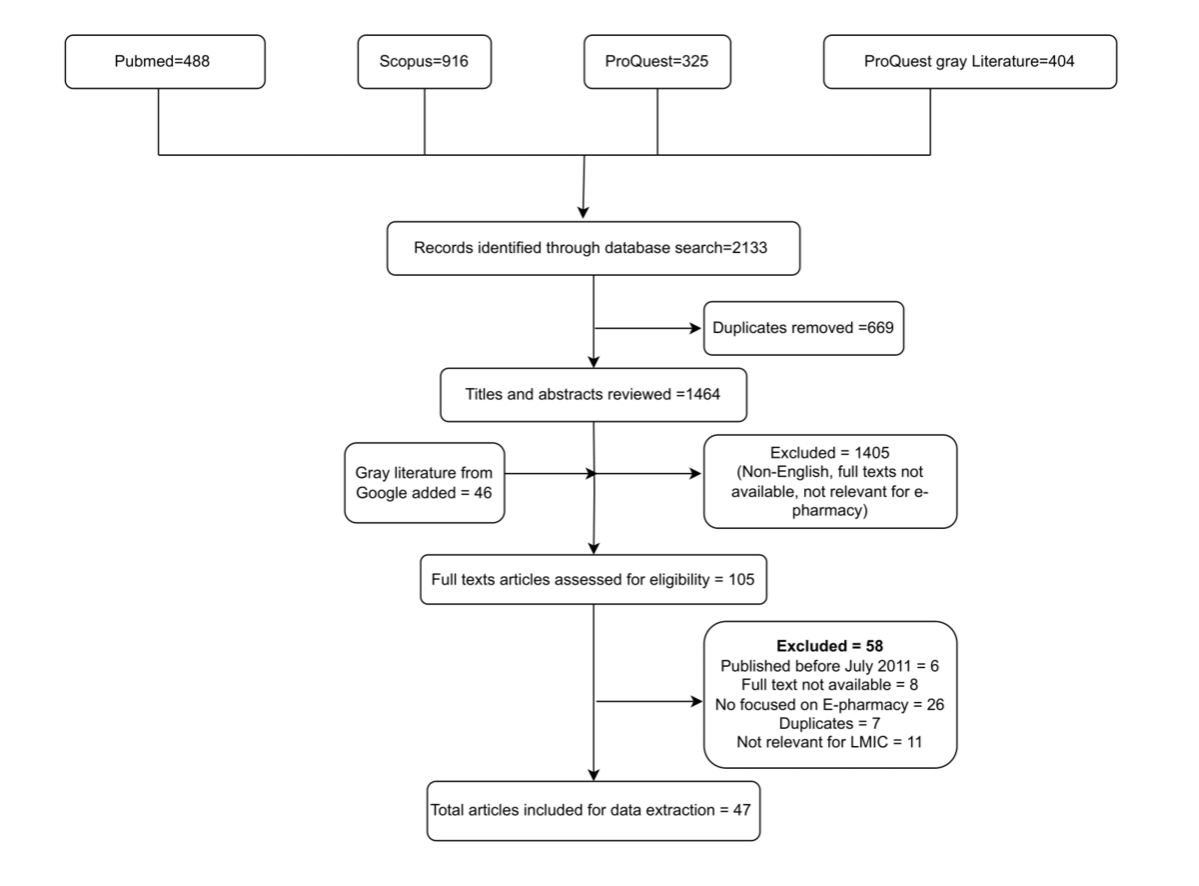
PRISMA (Preferred Reporting Items for Systematic Reviews and Meta-Analyses) flow diagram.

The search yielded 2133 records from MEDLINE, Scopus, ProQuest, and ProQuest Gray literature. After removing 669 duplicates, 1464 abstracts were screened for inclusion in the review. Of these, 47 full-text articles were included in the scoping review (Figure 1). These included 13 original papers, 11 literature reviews, 5 newspaper articles, 6 newsletters, 3 guidelines or reports from nongovernment organizations, 3 policy documents or government guidelines, 1 case study, 1 viewpoint, 1 PowerPoint presentation, 1 systematic review, and 2 blogs. Of these 47 articles, 32 were focused on India. In addition, 5 were focused on other Asian countries (China, Bangladesh, Taiwan, and Saudi Arabia).

### Study Characteristics

The study characteristics for original papers are provided in Table 1, which mainly comprised surveys done among consumers or pharmacists and observational studies using e-pharmacy websites. Table S2 in Multimedia Appendix 2 shows a summary of findings from other papers, including literature reviews, reports, guidelines, web pages, and newspaper articles.

**Table 1:** Study characteristics of the original studies included in the scoping review.

Author (year)	Country	Methods	Sample size	Findings
Jain et al (2019) [14]	India	Survey	80	Good awareness of online pharmacies; however, there is a chance of delivering wrong medicines and delayed delivery; there is less chance of unavailability of medicines.
Miller et al (2021) [11]	Kenya, India, and Nigeria	Literature review and key informant interviews	18 key informant interviews	None of the study countries had yet enacted a regulatory framework for e-pharmacy; Key regulatory challenges included the lack of consensus on regulatory models, lack of regulatory capacity, regulating sales across borders, and risks of overregulation.
Mulani et al (2018) [15]	India	Literature review and self-administered survey	100 retail pharmacists	All respondents were aware of e-pharmacy in India, and most of them expressed a positive attitude toward it.
Srivastava and Raina (2020) [16]	India	e-survey	184 consumers	Performance expectancy, effort expectancy, social influence, and hedonic motivation had a positive correlation with e-pharmacy adoption and the intention to recommend. Gender and educational background did not correlate.
Aithal and Shabaraya (2019) [17]	India	e-survey	258 (general public)	91% of the respondents purchased medicines offline almost always and 46% of respondents were happy with offline medicine purchases; 32.63% of the respondents who also purchase medicines online feel that attractive discounts on prices and offers are major factors in their decision.
Sabbir et al (2020) [18]	Bangladesh	e-survey	285 consumers	Perceived trust and health literacy were found significant in determining consumers’ intention to adopt e-pharmacy, while perceived risk and innovativeness were found insignificant.
Yan-Kwang et al (2019) [19]	Taiwan	Development of a sustainable inventory model	N/A^a^	This study proposes a single-period visual-attention-dependent demand (VADD) inventory model for e-pharmacies to cope with the sales growth of online marketing while reducing the cost of excessive inventory.
Yin et al (2016) [20]	China	Survey	274 consumers	Performance expectancy, social influence, perceived trust, and perceived risk have directly significant influences on consumers’ adoption intention of online medicine purchases.
Ma (2021) [21]	China	Survey	459 nonadopters of online pharmacy	Perceived usefulness and trustworthiness significantly affected nonadopters intention; perceived ease of use positively impacted perceived usefulness and trustworthiness; perceived risk negatively affected trustworthiness.
Brijnath et al (2014) [22]	Australia	Semistructured face-to-face interviews	28 Indian-Australians and 30 Anglo-Australians	Cost, convenience, and dissatisfaction with local health services were the main reasons for buying medicines online. Increased availability of medicines online raises important issues for the safe use of medicines.
Fittler et al (2013) [23]	Hungary	Observational study	136 online pharmacy websites	Only 41.2% of included websites were operational for up to 4 years; 44.1% were rogue, and 16.9% were unapproved; long-term continuous operation strongly correlated with explicit illegal activity.
Alwon et al (2015) [24]	United Kingdom	Observational study	113 websites selling diazepam, fluoxetine, and simvastatin	Less than a quarter of the websites were regulated; prescription was not mandatory in 80 websites; unregulated websites were found to adhere more closely to the clinical criteria and less likely to disclose the identity.
Bate et al (2014) [25]	United States	Email survey	2907 members of RxRights	61.54% purchase drugs online and mostly from foreign websites, citing cost savings as the leading reason.

^a^N/A: not applicable.

The results are described under the following subthemes.

### Regulations for e-Pharmacy in India

Out of 47 publications, 22 included information about regulations on e-pharmacy, and of these, 17 articles were written in the context of Indian settings (Table 1 and Table S2 in Multimedia Appendix 2).

In India, the Drugs and Cosmetics Act (1940), the Drugs and Cosmetic Rules (1945) [26], and the Pharmacy Act (1948) [27] are inadequate for the regulation of e-pharmacies [1]. Hence, a subcommittee of the Drugs Consultative Committee of the Drug Controller General of India (DCGI) suggested a draft amendment to the Drugs and Cosmetic Act, of 1945 (draft general safety regulation (GSR) from the Ministry of Health and Family Welfare, Government of India dated August 20, 2018) [1]. As per this amendment, for e-pharmacy, compliance with the Information Technology Act, 2000 [28] is required in addition to the 1945 rules.

As per these draft rules, the e-pharmacy needs to be registered with the DCGI, and the e-pharmacy portal should mention the registration details, official logo, the name of the registered pharmacist with the registration number, contact numbers, and return policy of dispensed medicines, details of the logistic service provider for the e-pharmacy. The premises or facility for the e-pharmacy business can be audited once in 2 years by the central licensing authority as per these rules [1]. In addition, the e-pharmacy portals should maintain the confidentiality of all the customer information except for regulatory audits and should not advertise in any print or digital media. Data thus collected should not be shared or stored outside the country [1,12]. There is no provision in the draft rules for the consumers to verify the authenticity of e-pharmacies. The sale of narcotics and psychotropics, tranquilizers, and Schedule X medicines through e-pharmacy is prohibited.

However, implementation of the draft rules has been pending for more than 5 years now. There is strong opposition to the growth of the e-pharmacy model in India by brick-and-mortar pharmacies. This is due to discrepancies in the rules for both and possibly because of vested interests in preventing the growth of e-pharmacies [11]. There have been public interest litigations in the Delhi and Madras high courts stating that in the absence of a monitoring system, the online sale of medicines can pose risks to doctors and patients [29]. The white paper published by the Indian Medical Association in 2015 opposed e-pharmacy due to the increased risk of drug abuse and drug misuse, including indiscriminate use of antibiotics and self-medication, as well as the proliferation of illicit pharmacies. Some of these concerns have been addressed in the current draft rules. However, the following issues need further action: the e-pharmacy portals can be used by children who cannot legally purchase medicines from a pharmacist; there is no mechanism to check the storage conditions of the medicines sold by e-pharmacies; mechanisms for recall of drugs sold through e-pharmacy are unclear [30]. With the increasing use of e-pharmacy in the country, online pharmacy retailers have come together to establish an association, the Indian Internet Pharmacy Association (IIPA). The association aims to protect and promote public health by maintaining harmony with regulators regarding the implementation of e-pharmacy in India. The IIPA is now renamed as Digital Health Platform (DHP) and is currently working actively with the Government to bring in changes to the regulations, including the use of Aadhar-linked prescriptions to appropriate use of e-pharmacy [31].

Despite these limitations, the e-pharmacy sector has grown by leaps and bounds in the last few years, especially with the advent of the COVID-19 pandemic, where doorstep delivery of medicines has been considered essential. Due to this, the Union Ministry, on March 24, 2020, mentioned pharmaceutical products as essential goods and delivery of medicines as essential services [32]. Recently, the department-related Parliamentary Standing Committee on Commerce Ministry, in its report presented to the Chairman of Rajya Sabha on June 15, 2022, recommended that the central government, in its draft e-pharmacy rules, should be finalized without any further delay. The committee expressed displeasure over the undue delay in the finalization of these rules, which is not conducive to the fast-paced digital markets [33].

Other regulatory challenges for the implementation of e-pharmacy in India include a lack of qualified personnel in the regulatory system, understaffing, corruption, and a lack of expertise to monitor online transactions. The e-pharmacy markets often operate beyond national borders, and regulators have no control over the purchase of medicines from pharmacies operating outside the country. Despite the presence of the rule that a doctor’s prescription must accompany a medicine, this rule is rarely enforced [11]. Furthermore, there is a lack of specific regulations for the sale of herbal medicines, which are easily available through the internet without a prescription and are often consumed under the popular belief that herbal drugs do not have adverse effects [34].

### e-Pharmacy and Access to Medicines

Access to medicines was mentioned in 14 publications included in the review. Except for 1 qualitative study, all publications were either reviews, blog posts, opinions, or reports (Table 1 and Table S2 in Multimedia Appendix 2). In one of the studies, which was conducted in Australia, patients (including Indo-Australians) taking antidepressants reported easy access through e-pharmacies to complementary and alternative medicines that were either not available or quite expensive in conventional pharmacies [22]. However, this was not the case with allopathic medicines, which were heavily subsidized and easily accessible from conventional pharmacies through the Pharmaceutical Benefits Scheme (PBS) in Australia.

The original evidence is scarce on what impact e-pharmacies may have on access to medicines in India. Nevertheless, there were a few studies on important facets of the access issue [35]. A narrative review highlighted the wide disparity in access to medicines by geographic area in India, probably due to the lack of conventional pharmacies and pharmacists in many parts. Although the distribution time in remote areas is delayed, certain e-pharmacies are known to reach and serve at least 90% of pin codes in the country. According to a report, e-pharmacies can aggregate supplies, making otherwise hard-to-find medicines available to patients across the country [5,36]. However, for an internet-naïve vulnerable population residing in remote areas with limited internet access, Deepika et al [37] believe that e-pharmacies may not increase access to medicines. Presently, internet penetration in India, which is essential for e-pharmacy ordering, is relatively low at 50% with better access in the urban as compared with rural areas [38]. According to the National Sample Survey Office’s 75th round national survey (2017-2018), only 8.5% of women in rural India can use the internet as compared with 17.1% of males. For urban areas, the percentage of internet users is significantly higher [39].

In urban India, e-pharmacies may help medicines reach the older adult population in particular. This group is often isolated due to the increasing adoption of the nuclear family structure. However, despite the convenience offered by e-pharmacies, there is considerable resistance from consumers in India [5,32]. A recent review reported that only 6% of respondents purchased medicines online, although more than 85% knew about e-pharmacies [14]. An expert opinion recommended that e-pharmacies establish logistics channels to reach out to patients in remote areas with less medical access [40]. It has also been suggested that the government should encourage generics to ensure the reach of medicines in remote areas [36].

### Quality of e-Pharmacy Services and Supply Chain-Related Issues

Quality of service or supply chain issues as well as products were studied or discussed in 6 original papers and 6 other publications (Table 1 and Table S2 in Multimedia Appendix 2).

A 2011 global systematic review found that only 12%-28% of e-pharmacies had at least 1 quality certification, and up to 17% of e-pharmacies did not have a secure website [41]. A global study on the quality of e-pharmacies found that 41% of e-pharmacies were in continuous operation for the 4-year study period [23]. However, 30% of the long-lived e-pharmacies were unapproved, and 64% were rogue. Though the physical location was disclosed by 54% of e-pharmacies, only 10% of e-pharmacies operated their server within the borders of the given location. Consumers were required to produce prescriptions and information on health status by 6% and 62% of e-pharmacies, respectively. These findings corroborate with another similar study from the United Kingdom published a year later [24].

Globally, the quality of e-pharmacy services is regulated using verification standards and professional certifications [23-25] by bodies such as the General Pharmaceutical Council (GPhC) in the United Kingdon and the National Association of Boards of Pharmacy (NABP) in the United States [42]. e-Pharmacies in the European Union (EU) should obtain a common logo that is displayed on their respective websites. Tapping on the logo would lead to a page where the authenticity of respective e-pharmacies can be verified [43]. Third-party certification agencies such as LegitScript accredit e-pharmacies worldwide and are endorsed by several regulatory authorities across the world.

India currently lacks any accreditation or certification body required to ensure the safety and health of consumers. The evidence comparing the quality of medicines purchased from e-pharmacies and conventional pharmacies is lacking in India. Certain e-pharmacies in India offer discounts, loyalty programs, and cashback to retain patients, and this effort may have an impact on the quality of medicines supplied or services provided [40]. Public perception of the quality of products supplied by e-pharmacies in India varies. In an e-survey, 26% of respondents believed that the quality of medicines would be compromised if medicines were traded online [5]. Conversely, in another survey, relatively more respondents believed that the quality of products supplied by e-pharmacies is better than that of conventional pharmacies [44].

Green supply chain management is increasingly considered globally and is adopted in Indian e-pharmacies [14,19]. E-pharmacies can reach out to local medicine stores for partnerships to cancel out supply chain issues in remote areas [36]. With certain e-pharmacies, medicines can be traced back to the channel, manufacturer, or supplier enabling quality checks [32]. Frost and Sullivan [12] suggest that substandard and fake medicines could be sold through conventional pharmacies, whereas e-pharmacies could prevent the sale of poor-quality medicines through their efficient tracking mechanism. However, as highlighted before, there are instances of suboptimal supply through e-pharmacy [36,45].

### Cost and Affordability of Pharmacy Services Using e-Pharmacy

Several published papers indicate that e-pharmacies can offer medicines at a lower cost as compared to conventional pharmacies (Table 1 and Table S2 in Multimedia Appendix 2). An e-pharmacy does not require a physical store for selling its merchandise and thus saves on both infrastructure costs and recurring costs like electricity and maintenance. This would result in significant cost reduction as most large pharmacies need to operate 12 hours a day, incurring huge maintenance and electrical costs. There is a cost involved in maintaining the infrastructure that consists of the hardware and software that run the e-pharmacy, shipping, and delivery costs. The reduced procurement and transactional costs are passed onto consumers at lower prices [8,40]. A study from the United States found that the cost of purchasing medications online for Parkinson disease ranged from 7% to 58% less for brand names and 31% to 76% less for generic medications compared with the community pharmacy [6,23].

A survey from India reports that 32.63% of respondents who purchase medicines online feel that discounts on prices and offers are major attractive factors for the use of e-pharmacy [17]. However, there are no studies from India comparing the cost-effectiveness of e-pharmacies.

### Advantages and Facilitators of e-Pharmacy

Global literature shows several advantages of e-pharmacies over brick-and-mortar pharmacies, such as improved access and reduced cost [46]. However, although the access is round-the-clock for the patients, the benefits offered by the round-the-clock access are not comparable with 24-hour brick-and-mortar pharmacies as the patients have to wait for a few hours or days till the medicines arrive [47]. Central stocks and digital accountability of stocks of e-pharmacy facilitate tracking of the supply chain and ensure easier access as compared with retail pharmacies [12,14,19]. Patients perceive less intimidation or embarrassment when they order medicines online rather than speaking directly to a pharmacist in a busy community pharmacy [47]. The anonymity offered by the internet encourages patients to seek information about medicines that they would otherwise avoid asking their physician or at an offline pharmacy, particularly for contraceptives, psychiatric diseases, erectile dysfunction, acne, sexually transmitted diseases, hair loss, etc. In a review of more credible Internet pharmacies, 1 study found that the quality of medical information was variable but generally more comprehensive than that provided by conventional pharmacy drugstores [48]. The e-pharmacies also offer cost comparisons across the internet databases thus empowering the consumers.

Advantages for the regulators and health system include the audit trail for all transactions including all details of medicines, prescribers, and e-pharmacy holders. These datasets can be used to inform public health policy; with increased transparency of transactions, 100% payment of applicable government taxes can be facilitated [5,11] and also facilitate back-tracing the channel or manufacturer or supplier of counterfeit and falsified medicines, thereby making the market a lot more transparent and authentic [46].

Recent literature shows good acceptance of e-pharmacy by users and physicians. In a survey by the Federation of Indian Chamber of Commerce and Industry (FICCI), almost 90% of Indian physicians perceived it as an acceptable means of sale and purchase of pharmaceutical products. The easy access and convenience of e-pharmacies are major determinants for attracting consumers in the view of medical practitioners, as mentioned by 85% and 75% of respondents, respectively [5]. A systematic review of e-pharmacy services in community pharmacy settings showed no difference in medication safety and adherence, conflicting evidence on patient satisfaction, and inadequate evidence on inappropriate medication use [49]. Recent literature shows that the use of e-pharmacy during COVID-19 was associated with a positive perception among pharmacists [50], was beneficial, especially in settings with a shortage of pharmacists [51], and could even reduce hospitalization due to COVID-19 [52].

### Disadvantages and Barriers to e-Pharmacy in India

Due to the absence of a physical structure and poorly evolved regulations, especially in the LMICs, including India, rogue pharmacies may outnumber legitimate pharmacies. Dispensing of substandard, falsified, or counterfeit medicines has been reported from such pharmacies due to a lack of oversight in e-pharmacy supply chains, and this may, at times, pose a life risk for patients [8]. Such pharmacies may allow the sale of medicines, including schedule-H medicines without a valid prescription. With the availability of health information on the internet, patients often self-diagnose and can get attracted by rogue e-pharmacies to self-medicate [10,53]. For example, toxicity associated with the self-administration of Zolpidem has been reported in the United Kingdom [45]. Although rogue pharmacies can prevail in brick-and-mortar pharmacies, the risk increases multifold with e-pharmacies as, in the absence of proper regulations, these can be run without the presence of a physical structure and qualified pharmacist and are thus less likely to get monitored. Studies conducted on the sale of psychiatric medicines found that patients with mental illness used online pharmacies to stock medicines believed to be effective in managing their condition. In addition, even when a prescription is available for the medicines, the consumer might still misuse it by sending a single prescription to multiple online pharmacies [54]. Additional disadvantages include delays in the delivery of medicines, lack of confidentiality, and medical supervision [55]. In a recent survey conducted among the general population in Chandigarh, India, most of the respondents preferred to consult their physicians before buying a drug online [56].

The pros and cons associated with e-pharmacy in the Indian context have been summarized in Table 2.

**Table 2:** Pros and cons of e-pharmacy in India.

Pros	Cons
Improved access: Available 24 hours a day, seven days a week.	No patient-pharmacist interaction: Lack of medical supervision or availability of registered pharmacists to answer essential questions through questions^a^.
The convenience of doorstep delivery: important for debilitated and older people, useful in pandemic conditions.	Risk of getting counterfeit, substandard, or incorrect medicines.
Offers anonymity to patients: Less intimidation while obtaining drugs for embarrassing health conditions^a^.	Concerns about privacy and confidentiality of personal information^a^.
Availability of drug-related information on the website^a^.	It may not be feasible for technology-naïve or illiterate people^a^.
Price comparisons are easy with searchable databases^a^.	Risk of misuse especially by using rogue pharmacies leading to adverse drug reactions or substance abuse.
Medicines can be offered at a considerably low cost.	Many rogue pharmacies provide drugs without valid prescriptions.
Can provide better access to medicines in remote and rural areas.	Lack of a national accreditation body to distinguish between authentic and rogue pharmacies^a^.
Potentially beneficial for chronic diseases to improve compliance with medicines^a^.	Lack of awareness about e-pharmacies among physicians and the general public^a^.
Ensured drug availability due to central stocks.	Limited participation by third-party payers.

^a^These factors are specific to e-pharmacies.

## Discussion

### Principal Findings

The present review summarizes available evidence from published and gray literature related to the implementation of e-pharmacy in India. The review shows clear benefits of e-pharmacy introduction in India in terms of improved access and delivery of medicines, especially in remote areas, for the older adult population and reduced costs of medicines. This is especially important in Indian settings considering the diverse pharmacist-patient ratios across various states [35] and the fact that medicines account for 70% of the health care costs in India [6]. E-pharmacy can also provide transparency in drug purchase and audit purposes. With the increasing use of e-pharmacy, to integrate clinical skills with the virtual environment, it has been suggested adding tele-pharmacy to pharmacy education [57]. Gil-Candel et al [58] showed that it is feasible to develop an e-pharmacy program within an outpatient pharmacy department of a tertiary hospital with home delivery of medicines.

The lack of clear regulations for e-pharmacy in India, lack of internet penetration, and digital literacy were found to be some of the important barriers to implementation. To combat the issue of internet connectivity and digital literacy, the Government of India has set up Common Services Centers (CSC) across the country to deliver the government’s e-services to rural and remote locations. According to the report, the Department of Pharmaceuticals aims to use CSCs for offering e-pharmacy services such as Jan Aushadhi medicines (generic medicines made available through the national program Bharatiya Jan Aushadhi Pariyojana). Patients may also search for and order their medications from Jan Aushadhi stores using the mobile or web-based applications that are being developed [5].

Specific regulation for e-pharmacy is a key step to propagating future investment and growth of e-pharmacy in India and curbing the unregulated growth of rogue pharmacies across the country. The introduction of these laws is possibly delayed as a result of hostility and litigations by retail pharmacist associations in the country [1]. The absence of comprehensive regulations on e-pharmacy in India is a possible loophole for selling substandard and counterfeit medicines [59]. An approved regulatory framework for e-pharmacy is keenly awaited to prevent confusion among the stakeholders and to increase investment in this sector. To expedite and smoothen the implementation of laws for e-pharmacy, policy makers should initiate dialogue with key stakeholders such as major pharmaceutical companies, IT companies, pharmacists, and physician associations. This can also help to resolve the issues responsible for the delay and thereby ensure the forward movement of the implementation of e-pharmacy in the country.

In addition, a national accreditation system should be in place to weed out rogue pharmacies, having robust technology solutions to interrupt the financial transactions of illicit pharmacies. As suggested by Miller et al [11], the presence of a global regulatory network may be the next step for the regulation of e-pharmacies due to the risk of the sale of medicines across national borders.

Improved literacy and awareness among consumers and health care providers about the use of legitimate pharmacies has been suggested to channel the e-pharmacy growth, and physicians should educate their patients about the dos and don’ts of online purchase of medicines [12,24].

**Figure 2:**
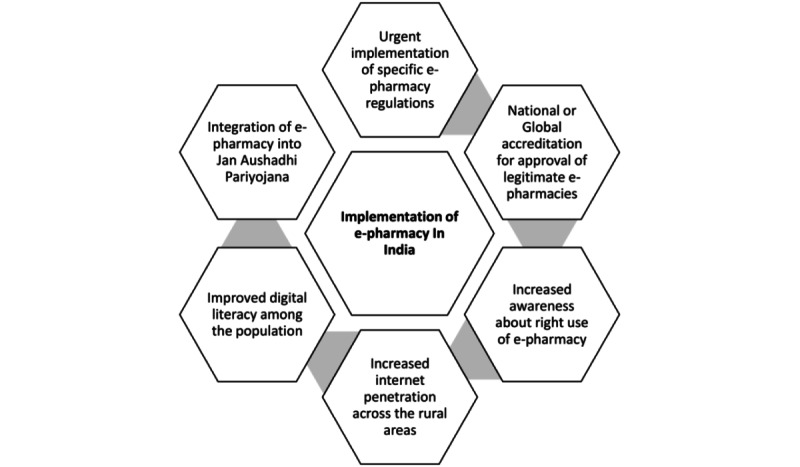
Key steps in the implementation of e-pharmacy in India.

The key steps in facilitating the implementation of e-pharmacies in India have been summarized in Figure 2.

The findings of our review are similar to recently published reviews on e-pharmacy from developing countries. A review of the effectiveness of e-pharmacy in rural Africa shows that e-pharmacy can improve health care access; lack of infrastructure, inadequate funding, and regulatory challenges are important hurdles in the implementation [60].

The review has certain limitations. We have used global literature with reference to the Indian context due to the scarcity of published literature. Furthermore, the gray literature including newsletters or data blogs is not peer-reviewed. In addition, due to delays in the peer review process, the review fails to include the most recent literature. Nonetheless, the review provides a consolidated overview of the status of e-pharmacy in India and key recommendations for the policy makers to consider during the implementation of e-pharmacy in India.

### Conclusion

Overall, e-pharmacy has the potential to improve access and affordability of medicines for Indian consumers by unleashing the power of technology for better health. However, there is an urgent need to implement a strong regulatory and accreditation network for the implementation of e-pharmacies in India to provide quality services and products through this model. In addition, increased internet penetration, improved digital literacy, raised awareness about legitimate e-pharmacies among doctors and patients, and integration into the public health program of Jan Aushadhi Yojana are needed to reap the real benefits of this technology.

## Appendix

Multimedia Appendix 1 Key themes and search strategies.

Multimedia Appendix 2 Summary of literature reviews and gray literature included in the scoping review.

Multimedia Appendix 3 PRISMA ScR (Preferred Reporting Items for Systematic Reviews and Meta-Analyses extension for Scoping Reviews) checklist.

## Abbreviations

CSC: Common Services Centers
DCGI: Drug Controller General of India
DHP: Digital Health Platform
EU: European Union
FICCI: Federation of Indian Chamber of Commerce and Industry
GSR: general safety regulation
GPhC: General Pharmaceutical Council
IIPA: Indian Internet Pharmacy Association
JBI: Joanna Briggs Institute
LMIC: low- and middle-income country
NABP: National Association of Boards of Pharmacy
OSF: Open Science Framework
PBS: Pharmaceutical Benefits Scheme
PRISMA-ScR: Preferred Reporting Items for Systematic Reviews and Meta-Analyses extension for Scoping Reviews
