# Supplementary material for: Facilitators, Barriers, and Potential Impacts of Implementation of e-Pharmacy in India and its Potential Impact on Cost, Quality, and Access to Medicines: Scoping Review
Source: Online J Public Health Inform. 2024 Oct 9;16:e51080. doi: 10.2196/51080 (PMC11499724; doi:10.2196/51080)
Supplement: Multimedia Appendix 1 [file ojphi_v16i1e51080_app1.docx]

**Table S1:** Key themes and search strategies.

| **Sr No** | **Theme** | **Search strategy** |
| --- | --- | --- |
| #1 | E-pharmacy | E-pharmacy, Online Pharmacy, Internet Pharmacy, Digital Pharmacy, Tele-pharmacy, Online Drug/s, Online Medicine/s, Pharmacy website/s, Pharmacy mobile application, Electronic prescription, Electronic dispensing, Online pills, E-prescription |
| #2 | Facilitators and barriers | Advantage/s, Determinant/s, Challenge/s, Disadvantage/s, Obstacle/s, Block/s, Impediment/s, Limitation/s, Difficulty /difficulties, Problem/s, Facilitator/s, Barrier/s, Enabler/s, Factor/s, Influencer/s, Feasibility/feasible |
| #3 | Access | Approachability/approachable, Availability/Available, Ease of use, Access |
| #4 | Quality | Quality, Delivery, Supply chain, Safety, Stock, Inventory, Management, Correctness, Precision, Time |
| #5 | Cost | Cost, Affordability, Economic, Finance/financial, Cheap, Expensive, Inexpensive |
| #6 | Regulations | Act, Rules, Guidelines, Law, Schedules |
|  | Final search strategy | #1 [title] AND (#2 OR #3 OR #4 OR #5 OR #6)[title/abstract] |
