# Supplementary material for: Facilitators, Barriers, and Potential Impacts of Implementation of e-Pharmacy in India and its Potential Impact on Cost, Quality, and Access to Medicines: Scoping Review
Source: Online J Public Health Inform. 2024 Oct 9;16:e51080. doi: 10.2196/51080 (PMC11499724; doi:10.2196/51080)
Supplement: Multimedia Appendix 2 [file ojphi_v16i1e51080_app2.docx]

**Table S2: Summary of literature reviews and grey literature included in the scoping review**

| **Sr No** | **Type of article** | **Ref** | **Year** | **Country** | **Focus group** | **Findings** |
| --- | --- | --- | --- | --- | --- | --- |
| 1 | Newsletter | (1) | 2017 | United Kingdom | Patients/Consumers | Although online medical consultations increase healthcare access and increase convenience, it create complex issues regarding medication safety. The model of online shopping is not appropriate for the complexity of delivering safe and effective healthcare. |
| 2 | Literature review | (2) | 2020 | Saudi Arabia | Patients/Consumers, Pharmacists | Trust and risk are critical factors leading to the users' intention to adopt the system. It is therefore recommended that building confidence in ensuring the safety of the medium will minimize the risk perception of the users. The findings of this study are believed to be of great benefit to online pharmacy stores in Saudi Arabia. |
| 3. | Literature review | (3) | 2019 | India | Patients/Consumers, regulators | This article represents, the sudden consequences towards e-pharmacy and its validated growth firms on Herbal and Organic products with a comparison of offline and online medical stores –Consumers need to be educated about the need to verify the authenticity of the service provider as well as the product.  A mutual awareness of online purchases of medicines by the patient and consumer is necessary for better management and avoidance of the consequences of self-medication. We need to reflect on the consumers’ interests and inclinations for online pharmacies to enhance a symbiotic physician-pharmacist-patient relationship. These measures coupled with adequate monitoring from regulators can help the consumer reap the rich benefits of these pharmacies. |
| 4. | Literature review | (4) | 2020 | India | Patients/Consumers, pharmacists, regulators | E-Drug stores are likely to increase self-medication, medication misuse, chronic drug use, and biased prescribing in the clinical sector. This can further increase non-compliance, drug resistance, prolong hospitalisation & increase in morbidity and mortality among the patients. So due to the rise in popularity of the e-pharmacy concept in India, we can expect to see the finalisation of proposed norms and some relevant amendments to the existing laws governing pharmaceutical operations in India, soon |
| 5. | Literature review | (5) | 2016 | India | Patients/Consumers, pharmacists regulators | Consumer education to verify the authenticity of the service provider and the product. Good online pharmacies have well-defined safety and quality benchmarks, uncomplicated privacy and security policies, a verifiable physical address, and a licensed pharmacist on the roll. Clinicians should familiarize themselves with a few reputable and legitimate online pharmacies that they may recommend to their patients. Need to reflect on the consumers' interests and inclinations for online pharmacies to enhance a symbiotic physician-pharmacist-patient relationship. Adequate regulatory monitoring is required. |
| 6. | Literature review | (6) | 2016 | India | Regulators | Comparison of regulations for internet pharmacy across USA and UK. USA requires mandatory registration, accreditation by NABP, the existence of a physical store, and sale is regulated by 21CFR. UK – Registration with the general pharmaceutical council (GPhC). Pharmacists should be registered with GPhC, physical stores should be present in the UK. Sale regulated by MHRA. Both countries have clear policies laid down for the online sale of drugs. |
| 7. | Literature review | (7) | 2015 | United States | Pharmacists, regulators | The number of reputable online pharmacies is dwarfed by those that operate illegally. The creation of an international network for online pharmacies can be a solution. Unsafe websites: Do not provide contact information, offer dramatically low prices, dispense prescription-only medications without a valid order, medications of unknown quality or origin, incorrect medication, and no protection of personal health information. |
| 8. | Systematic review | (8) | 2011 | Global review | Patients/Consumers, regulations | Several concerns like lack of prescription requirements, access across the globe, lack of data privacy and personal protection, risk of getting sub-standard drug quality, and lack of transparency about the details of the online pharmacy were identified. The authors suggested 2 level approach – 1. Strengthen regulations for online pharmacy; 2. Improving the health literacy of the overall population. |
| 9. | Case study | (9) | 2016 | United Kingdom | Patients/Consumers, Physicians | Case study about the overdose of Zolpidem by an elderly gentleman without informing his general practitioner. Survey amongst psychiatric practitioners – lack of knowledge about online pharmacy. Physicians should warn the public about the possible risks and should actively ask patients about any online drug use. Also, patients should inform their practitioners about the same. |
| 10. | Viewpoint | (10) | 2020 | India | Patients/Consumers, regulators, pharmacists | Loopholes in the current pharmacy system – Weaker laws, Poor access and availability to Jan Aushadhi Yojana, dependence on private pharmacies, family members of registered pharmacists selling drugs, sale of schedule H, H1, and X drugs without a valid prescription. Benefits of E-pharmacy – for increasing the burden of non-communicable diseases the cost and access can be improved. A system similar to NABP/VPPS should be made available for oversight of E-pharmacies. |
| 11. | Literature review | (11) | 2016 | USA | Patients/Consumers, Pharmacists, physicians, regulators | Increased prevalence of illicitonline pharmacies Need technology solutions and international regulations to combat illicitonline pharmacies, a critical need to build international consensus on a coordinated multi-stakeholder response to address complex threats posed by illicit online pharmacies. |
| 12. | Newsletter | (12) | 2021 | India | Regulators, pharmacists, patients | Challenges of E-pharmacy in India - Absence of proper guidelines, lack of technical infrastructure, no awareness in the rural areas, and unfavourable delivery in times of emergency. Advantages – better access and lower cost. E-pharmacies may be the best option in the pandemic situation. |
| 13. | Newsletter | (13) | 2021 | India | Pharma companies, regulators | E-pharmacy growth drivers: increasing internet penetration, increasing e-commerce adoption, push to organized channels, changing disease profile. E-pharmacy is still in its early days with immense potential to scale and grow. |
| 14. | Newsletter | (14) | 2020 | India | Regulators, pharmacists | Unclear regulatory status on E-pharmacy in India, voluntary code of conduct by the Indian Internet Pharmacy Association to process medicines only against prescription from a registered  medical practitioner, not sell Schedule X drugs, and dispense medicines only through licensed pharmacies. Challenges - misuse of medical and other personal information, usage of fake prescriptions to avail drugs, supply of prescription drugs based on a nondescript online questionnaire. |
| 15. | Literature review | (15) | 2016 | India | Regulators, pharmacists | Three types of models of e-pharmacies exist in India: organized, non-organized and illegal. Major concerns – the risk of drug abuse, misuse, resistance, addiction due to pain killers, CNS depressants, etc, ambiguous regulations. E-pharmacies may affect doctor-pharmacist relations. |
| 16. | Newsletter | (16) | 2020 | India | Regulators, pharmacists | Existing laws - Information Technology Act, 2000; the Drug and Cosmetics Act, 1940; Drugs and Cosmetic Rules, 1945; Pharmacy Act, 1948; and the Indian Medical Act, 1956. govern the online pharmacies in India. Regulations are needed for the safe selling of lifestyle drugs, but checking the validity of prescriptions by online pharmacies is not entirely effective. Advantages – better access, less cost, and convenience of doorstep delivery, media alerts validation of prescription through the online pharmacist. |
| 17. | Newspaper article | (17) | 2019 | India | Pharma companies, physicians, pharmacists | Estimated E-pharmacy market of US $3,657 million (~Rs.25,000 crore) by 2022; can account for 15-20% of pharma sales in next 10 years, by enhancing adherence and access to medicines for a majority of the under-served population, active pharmacist counselling, long term drug compliance for chronic conditions, and cost savings on bills along with doctor consultation support. The absence of manufacture-level bulk packing and barcoding is a challenge. |
| 18. | Blogs | (18) | 2021 | India | Pharmacists, technology professionals | Challenges- Lack of trust due to counterfeit medicines, Limited customer support (chatbots, self-help systems, and faster payments and refunds), and limited presence in regional languages. E-pharmacies must tap into the rising logistics bandwidth in remote areas. Concerns about data privacy, stricter timebound deliveries, collaboration with hospital chains, and campaign towards boosting trust among the customers. Governing laws are much needed to flourish future growth. |
| 19. | Literature review | (19) | 2018 | India | Pharmacists, physicians, regulators, technology professionals | Pros – Ease of online ordering, the potential for integrating into Jan Aushadhi Yojana, time-saving, cost-saving, offers a range of choices, the confidentiality of patients is maintained, legal stores have a process for drug approval.  Cons – Lack of medical supervision, rogue pharmacies can provide drugs without prescriptions, safety risks, some sites may not maintain the privacy of consumers, and lack the personal touch that conventional pharmacies have. |
| 20. | Guidelines or reports | (20) | 2016 | India | Patients/Consumers, pharmacists, physicians, regulators | The implementation of the e-Pharmacy model needs to be based on four guiding principles:  · Orderly growth of e-Commerce in India  · Model that best serves the following consumer interests should be adopted:  – Patient Safety  – Proper Access to Medicines  – Authenticity  Recommendations for Due Diligence of the e-Pharmacy Model  - Physically Verifiable Address of the Seller, Separate License and Registry of the e-Pharmacies, the requirement of original prescription, audit trail, restriction for Habit-forming Drugs, the team of qualified pharmacists for validation of prescription, authentic and detailed information on the website with contact details, and the confidentiality of Patient Information: Internet-based transactions are well aligned to address key known issues in pharmacy retail for tracking authenticity, traceability of medicine, abuse prevention, addressing consumption of drugs without prescription. It may lead to the adoption of e-prescriptions by doctors in future. Huge economic benefit for the middle-class population. |
| 21. | Guidelines or reports | (21) | 2020 | India | Patients/Consumers, physicians, pharmacists, regulators, technology professionals | Awareness about E-pharmacies in 65% of consumers and 73% reported a positive impact. E-pharmacy is a building block for the digital health ecosystem, with clear tangible benefits to consumers – tax loss and value-added services, alignment with the national goals of universal affordable health coverage, E-pharmacy can stimulate transparency and price competition in the market, online retail of medicines from Janaushadhi Pariyojana centres, via the VLE network at CSCs, |
| 22. | Newspaper article | (22) | 2020 | India | Patients/Consumers, regulators | three business models — marketplace, inventory-led hybrid (offline/online) and franchise-led hybrid (offline/online) — depending on the way the supply chain is structured. The final notification for the draft rules for E-pharmacy is awaited. |
| 23. | Powerpoint presentations | (23) | 2019 | India | Pharmacists, physicians, technology professionals | e-Pharmacy is in a nascent stage in India, and the potential to be large industry segment soon, E-Pharmacies Evolving as a one-stop solution for all healthcare needs to improve the consumer database.  Recommendations: Valid prescription, personal supervision, of a registered pharmacist, audit trail, restriction for narcotic pills, suitable arrangements for packing of good quality medicines, the authentic website with all information, leveraging big data and machine learning. |
| 24. | Blogs | (24) | 2021 | India | Pharmacists, regulators | Licenses required: Retail Drug License, GST number. Before setting up your online pharmacy website/app, you need to set up a physical pharmacy as in the case of a traditional pharmacy. |
| 25. | Guidelines or reports | (25) | 2020 | India | Pharmacists, physicians, regulators, technology professionals | Rise in the telemedicine services post-pandemic, E-pharmacies can offer integrated solutions, E-pharmacy purchases primarily driven by discounts and convenience, concerns about counterfeit medicines, data privacy and prescription abuse, the necessity of regulations which mandate consultation with the doctor before providing a substitution, concerns about substitution practices and their conflict with offline channels, discord between online and traditional channels. |
| 26. | Newsletter | (26) | 2019 | India | Pharmacists, physicians | Impact of e-prescription on e-pharmacy operations – save time. An amalgamation of e-pharmacy with e-prescriptions under the supervision of a registered pharmacist can modernize the pharmacy systems for the 21st Century. |
| 27. | Guidelines or reports | (27) | 2015 | India | Pharmacists, physicians, regulators | Opposition by Indian Medical Association is against online pharmacies- the risk of substitution of cheaper and spurious drugs by the online stores and the doctor-patient confidentiality will affected, can encourage patients to use one prescription repeatedly, risk of drug abuse and adverse effects, medicolegal liability will still fall on the doctor. E-pharmacy can be utilized by affluent and educated people, with no public health benefits. |
| 28. | Newspaper article | (28) | 2021 | India | Patients/Consumers, pharmacists | India’s online pharmacy sector is once again witnessing a surge in the second COVID wave. |
| 29. | Government report/policy document | (29) | 2021 | India | Patients/Consumers, regulators | The online purchase channel of medicines is an additional facility but not a substitution for a pharmacy. There is a need for proper regulations for the sale of packaging, sale, and safety standards. |
| 30. | Newspaper article | (30) | 2021 | India | Patients/Consumers, regulators | E-pharmacy technology can successfully implement Pradhan Mantri Bhartiya Janaushadhi Pariyojana (PMBJP) National Digital Health Mission. E-pharmacy can offer better-purchasing margins, better inventory management, increased reach, reduced prices, and greater provision of value-added services to the consumers. |
| 31. | Literature review | (31) | 2021 | India | Pharmacists, regulators | Indian e-pharmaceutical judiciary and regulatory authority should take into account the steps adopted by numerous other developing countries such as the USA and the EU with common logo labels, electronic monitoring programs, and e-prescriptions for some medicines to tackle problems related to the online selling of medication in India. |
| 32. | Newspaper article | (32) | 2020 | India | Patients/Consumers, regulators | some of India’s largest conglomerates and multinational e-commerce firms are looking to gain a foothold in the sector. Private firms are investing in E Pharmacy. The fledgling sector continues to be beset by legal challenges, in the absence of a clear regulatory framework from India's policymakers. |
| 33. | Government report/policy document | (33) | 2018 | India | Regulators, pharmacists | Procedure for distribution or sale, of drugs through e-Pharmacy, - On receipt of prescription, through the e-pharmacy portal, the registered pharmacist on behalf of the e-pharmacy registration holder shall verify the details of the patient, Registered Medical Practitioner, and arrange for the dispense of the drugs as per the instructions of the Registered Medical Practitioner, if not already found dispensed;  (2) The e-pharmacy registration holder who has received a prescription in sub-rule (1) shall dispense and made arrangement for the supply of drugs from any retail or wholesale licensed premises under the Drugs and Cosmetics Act, 1940 and rules made thereunder;  (3 The details of the drugs dispensed including the patient details shall be maintained on the e-pharmacy portal.  (4) In the case of e-prescription, the prescription shall be uploaded on the e-pharmacy portal and shall be kept in the record by the dispenser. Prohibition of advertisement of drugs through e-pharmacy, - No e-pharmacy shall advertise any drug on radio or television or internet or print, or any other media for any purpose. |
| 34. | Literature review | (34) | 2011 | India | Regulator, pharmacist | Improved patient safety / better patient health outcomes; - Standardisation of data, information exchange, and business processes; Improved and timely monitoring, tracking, and reporting. Key to the success of e-pharmacy is cooperation from physicians in creating electronic health records of patients and the IT hardware and software support system for making it possible to share information, financial incentives for physicians, and the spread of health insurance. Many states in India do not have an adequate pharmacist-patient ratio. The ideal ratio is 1:2000 as per WHO recommendations. |

**References:**

1. What price drugs online? Drug Ther Bull [Internet]. 2017 Aug;55(8):85. Available from: https://www.proquest.com/scholarly-journals/what-price-drugs-online/docview/1928599976/se-2?accountid=37964

2. Alsadoun AA, Iskandar YHP, Tangiisuran B. Exploring the factors that influence the adoption of online pharmacy in saudi arabia: A conceptual review. Journal of Critical Reviews [Internet]. 2020;7(16):354–60. Available from: https://www.scopus.com/inward/record.uri?eid=2-s2.0-85087800157&doi=10.31838%2Fjcr.07.16.43&partnerID=40&md5=029fe9a03c8d2cf6ad1c0118c6ac2b30

3. Anbarasi M, Kumar SP. Consequences towards E-pharmacy and its validated growth firms on herbal and organic products. Indian J Public Health Res Dev [Internet]. 2019;10(8):150–5. Available from: https://www.scopus.com/inward/record.uri?eid=2-s2.0-85073519321&doi=10.5958%2F0976-5506.2019.01868.0&partnerID=40&md5=4b681a7e927cb02cd5537c0d5bd6da1d

4. Deepika, Singh R, Singh TG, Singh M, Saini B, Kaur R, et al. Status of E-pharmacies in India: A review. Plant Arch [Internet]. 2020;20:3763–7. Available from: https://www.scopus.com/inward/record.uri?eid=2-s2.0-85086152648&partnerID=40&md5=4bacd93335ec4b89a100daa26708bf0d

5. Desai C. Online pharmacies: A boon or bane? Indian J Pharmacol [Internet]. 2016;48(6). Available from: https://www.proquest.com/scholarly-journals/online-pharmacies-boon-bane/docview/1900902443/se-2?accountid=37964

6. Divekar R v, Gaud RS. A comparative study for internet pharmacy-sale of drugs in USA and UK. Int J Pharm Sci Rev Res. 2016;38(1):175–8.

7. Gabay M. Regulation of Internet Pharmacies: A Continuing Challenge. Hosp Pharm. 2015 Sep;50(8):681–2.

8. Orizio G, Merla A, Schulz PJ, Gelatti U. Quality of online pharmacies and websites selling prescription drugs: A systematic review. J Med Internet Res. 2011;13(3):1–42.

9. Rajagopal M. Internet pharmacies - boon or threat? Prog Neurol Psychiatry [Internet]. 2016;20(5):4–5. Available from: https://www.proquest.com/scholarly-journals/internet-pharmacies-boon-threat/docview/1831704430/se-2?accountid=37964

10. Satheesh G, Puthean S, Chaudhary V. E-pharmacies in India: Can they improve the pharmaceutical service delivery? J Glob Health. 2020;10(1):1–3.

11. Mackey TK, Nayyar G. Digital danger: a review of the global public health, patient safety and  cybersecurity threats posed by illicit online pharmacies. Br Med Bull. 2016 Jun;118(1):110–26.

12. Venkatachalam L, Origins. An Overview of the Online Pharmacy industry in India [Internet]. 2021 [cited 2022 Jun 8]. Available from: https://startuptalky.com/online-pharmacy-industry-india/

13. Us A, Us J, To S, Story G, Manufacturing B, Services C, et al. e-Pharmacies – Bridging the gap in Indian healthcare. 2021;1–13.

14. India : E-Pharmacies : The Story So Far & The Way Forward by. 2021;968136.

15. VP P, BK A. E-pharmacies Regulation in India: Bringing New Dimensions to Pharma Sector. Pharm Regul Aff. 2016;05(02):1–7.

16. Sanghi Kriti. E-pharmacies -Regulations in India [Internet]. [cited 2022 Jun 9]. Available from: https://samistilegal.in/e-pharmacies-regulations-in-india/

17. Kumar BT. ePharmacy - Global Trends and challenges that India presents, Health News, ET HealthWorld. 2022;1–11. Available from: https://health.economictimes.indiatimes.com/news/pharma/epharmacy-global-trends-and-challenges-that-india-presents/67866010

18. Winning E pharmacyindia I, Battle T, Traditional O, Winning E pharmacyindia I, Battle T, Traditional O. Commerce Dashboard IBEF : India Brand Equity Foundation. 2021;1–10.

19. v. Chordiya S, M. Garge B. E-pharmacy vs conventional pharmacy. IP International Journal of Comprehensive and Advanced Pharmacology. 2020;3(4):121–3.

20. Jaisani L, Pant S, Shivam K, FICCI. E-Pharmacy in India : Last Mile Access To Medicines. Indian National BAR Association [Internet]. 2016;80. Available from: https://www.indianbarassociation.org/wp-content/uploads/2016/08/E-Pharmacy-in-India-Last-Mile-Access-to-Medicines_v6.pdf

21. Consulting R. ePharmacies at COVID-19 Frontline. 2020;(August).

22. Act C. Explained : How India ’ s online pharmacy market is regulated. 2021;1–15.

23. Sullivan F&. In the spotlight: e-Pharmacy in India. An exponential growth opportunity. Frost & Sullivan [Internet]. 2019;1–52. Available from: https://ww2.frost.com/frost-perspectives/spotlight-e-pharmacy-india-exponential-growth-opportunity/

24. Kamboj BA. How to start E-Pharmacy Business in India? – Pharma Franchise Help [Internet]. 2021. p. 1–9. Available from: https://pharmafranchisehelp.com/how-to-start-online-pharmacy-sell-medicines-internet-drug/#RULES

25. Healthcare goes mobile: Evolution of teleconsultation and e-pharmacy in new Normal September 2020. 2020.

26. Mart P. Impact of e-prescription on e-pharmacy operations. 2021;9–10.

27. Indian Medical Association. IMA White Paper on Online Pharmacy. 2015;42(2):1–5. Available from: http://www.ima-india.org/ima/left-side-bar.php?scid=324

28. Peermohamed A, Shah S. Online pharmacy firms see massive order surge in second wave - The Economic Times. ETonline [Internet]. 2021;1–9. Available from: https://economictimes.indiatimes.com/tech/startups/online-pharmacy-firms-see-massive-order-surge-in-second-wave/articleshow/82394940.cms?from=mdr

29. Business P. Laws For Online Pharmacy Business In India The Central Drugs Standard Control Organization ( CDSCO ). 2021;2–5.

30. Article R. The government needs to notify the nal e-pharmacy rules at the earliest. 2021;1–7.

31. Ayush Verma. Regulation of e-pharmacies [Internet]. [cited 2022 Jun 10]. Available from: Regulation of e-pharmacies - iPleaders.html 2/9

32. 2020 Year in Review | How Covid-19 is reshaping online pharmacy in India [Internet]. [cited 2021 Jun 21]. Available from: https://m.economictimes.com/tech/startups/2020-year-in-review-how-covid-19-is-reshaping-online-pharmacy-in-india/articleshow/80010076.cms

33. Ministry of Health and Family Welfare G of I. GSR 817 dated August 20, 2018 [Internet]. Gazette of India. Available from: https://cdsco.gov.in/opencms/opencms/en/Notifications/Gazette-Notifications/

34. Bhatia S. Role of E-pharmacy in reducing accessibility dispersion between Indian states. In: 5th International Multi-Conference on Society, Cybernetics and Informatics, IMSCI 2011 [Internet]. Indian Institute of Foreign Trade, New Delhi, 110016, India: International Institute of Informatics and Systemics, IIIS; 2011. p. 264–7. Available from: https://www.scopus.com/inward/record.uri?eid=2-s2.0-84896258751&partnerID=40&md5=f702b67d865ec838ab51e53aed0c25a4
